# Conversion surgery for initially unresectable locally advanced pancreatic ductal adenocarcinoma after chemotherapy followed by carbon-ion radiotherapy: a case report

**DOI:** 10.1186/s13256-023-04311-3

**Published:** 2024-01-11

**Authors:** Yusuke Watanabe, Takaaki Tatsuguchi, Kenjiro Date, Tomohiko Shinkawa, Hirotaka Kuga, Sadafumi Tamiya, Kazuyoshi Nishihara, Toru Nakano

**Affiliations:** 1 Department of Surgery, Kitakyushu Municipal Medical Center, 2-1-1 Bashaku, Kokurakita-Ku, Kitakyushu, 802-0077 Japan; 2 Department of Pathology, Kitakyushu Municipal Medical Center, 2-1-1 Bashaku, Kokurakita-Ku, Kitakyushu, 802-0077 Japan; 3 Department of Surgery, Hamanomachi Hospital, 3-3-1 Nagahama, Chuo-Ku, Fukuoka, 810-8539 Japan

**Keywords:** Pancreatic ductal adenocarcinoma, Carbon-ion radiotherapy, Conversion surgery

## Abstract

**Background:**

Recent advances in chemotherapy and chemoradiotherapy have enabled conversion surgery (CS) to be performed for selected patients with initially unresectable locally advanced (LA) pancreatic ductal adenocarcinoma (PDAC). Many studies indicate CS might extend the survival of patients with initially unresectable LA PDAC. However, several clinical questions concerning CS remain, such as the optimal preoperative treatment. Carbon-ion radiotherapy (CIRT) is a unique radiotherapy that offers higher biological effectiveness than conventional radiotherapy. Here, we report a long-term survival case with initially unresectable LA PDAC who underwent CS after chemotherapy followed by CIRT.

**Case presentation:**

The patient was a 72-year-old Japanese woman with unresectable LA pancreatic head cancer with tumor contact to the superior mesenteric artery (SMA). She underwent four courses of chemotherapy (gemcitabine plus nanoparticle albumin-bound paclitaxel). However, the lesion did not shrink and tumor contact with the SMA did not improve after chemotherapy. Because the probability of achieving curative resection was judged to be low, she underwent radical dose CIRT, and chemotherapy was continued. She complained of vomiting 2 months after CIRT. Although imaging studies showed no tumor growth or metastasis, a duodenal obstruction which was speculated to be an adverse effect of CIRT was observed. She could not eat solid food and a trans-nasal feeding tube was inserted. Therapeutic intervention was required to enable enteral nutrition. We proposed several treatment options. She chose resection with the expectation of an anti-tumor effect of chemotherapy and CIRT rather than course observation with tube feeding or bypass surgery. Therefore, subtotal-stomach-preserving pancreatoduodenectomy with portal vein resection was performed as CS. Pathological examination of the resected specimen revealed an R0 resection with a histological response of Evans grade IIA. Postoperatively, she recovered uneventfully. Adjuvant chemotherapy with tegafur/gimeracil/oteracil (S1) was administrated. At the time of this report, 5 years have passed since the initial consultation and she has experienced no tumor recurrence.

**Conclusions:**

The present case suggests that multidisciplinary treatment consisting of a combination of recent chemotherapy and CIRT may be beneficial for unresectable LA PDAC. However, further studies are required to assess the true efficacy of this treatment strategy.

**Supplementary Information:**

The online version contains supplementary material available at 10.1186/s13256-023-04311-3.

## Background

The prognosis of pancreatic ductal adenocarcinoma (PDAC) remains dismal despite recent advances in multimodal treatment. Complete surgical resection of PDAC is the only potential hope of a cure and is the most relevant predictor of long-term survival [1, 2]. One of the reasons for the dismal prognosis of PDAC is that most diagnoses are made when the disease is either locally advanced (LA) or metastatic, and only 20% to 30% of patients have resectable PDAC at diagnosis [3, 4]. Recent advances in chemotherapy or chemoradiotherapy have enabled “conversion surgery (CS)” to be performed for selected patients with initially unresectable PDAC following favorable responses to preoperative treatment. The prognosis of patients who underwent CS for initially unresectable PDAC was reported to be more favorable than for those who could not undergo CS [5, 6]. However, there are many unresolved clinical questions concerning CS for initially unresectable PDAC, such as the optimal preoperative therapy, duration of preoperative therapy, and indication of patient selection for CS. Chemotherapy alone and chemoradiotherapy are both considered standard-of-care for unresectable LA PDAC [3]. However, the role of radiotherapy for unresectable LA PDAC is controversial. Carbon-ion radiotherapy (CIRT) is a unique external-beam radiotherapy that offers higher biological effectiveness than conventional radiotherapy, and recent impressive results of Japanese studies investigating CIRT for PDAC have attracted global attention [7, 8].

Here, we report a long-term survival case with initially unresectable LA PDAC who underwent CS after chemotherapy followed by CIRT.

## Case presentation

A 72-year-old Japanese woman was admitted to her family doctor with epigastric discomfort and weight loss (10 kg/year). Imaging studies suggested pancreatic head cancer; thus, the patient was referred to our department. Her weight at the time of initial consultation to our department was 48.9 kg (body mass index was 22.1 kg/m^2^). She had a medical history of hypertension and no family history of cancer. Laboratory data indicated a decreased hemoglobin concentration of 10.9 g/dL. The other laboratory data, including tumor marker concentrations (carcinoembryonic antigen (CEA) concentration of 2.8 ng/mL and carbohydrate antigen 19-9 (CA19-9) concentration of 15.6 U/mL), were within normal limits.

**Fig. 1:**
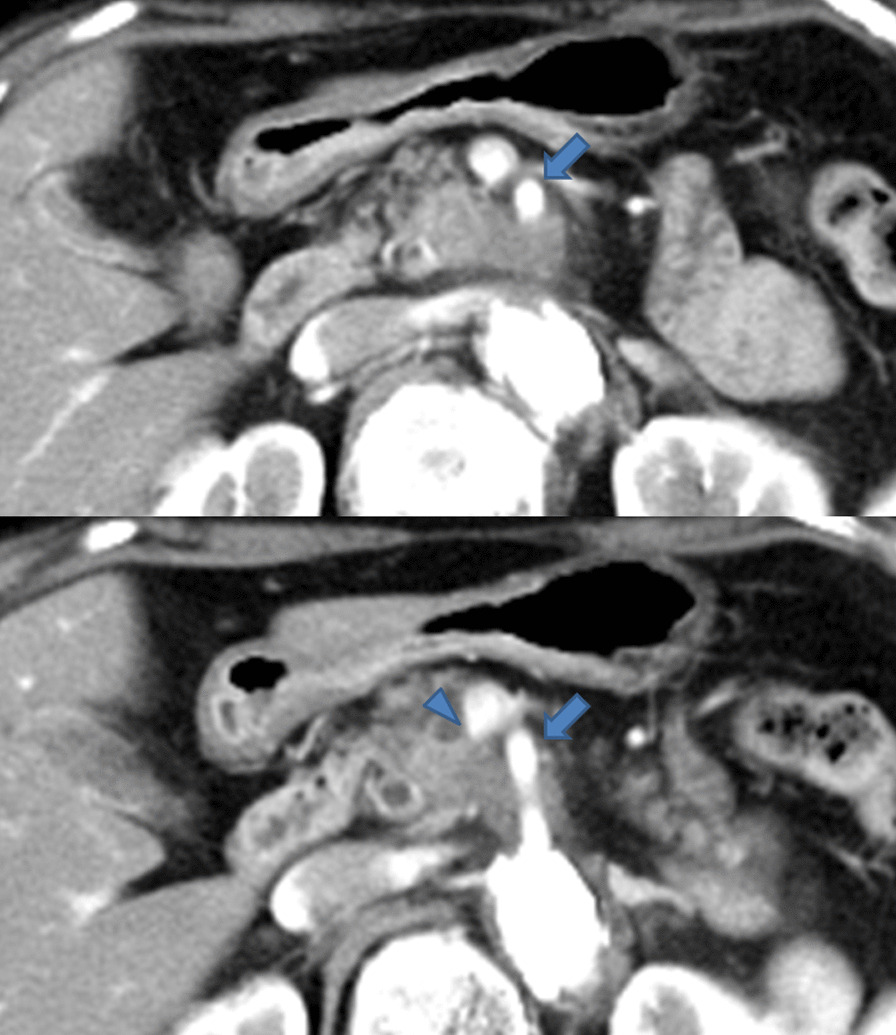
Contrast-enhanced computed tomography findings. CECT revealed a hypovascular tumor in the uncinate process of the pancreas with tumor contact to the superior mesenteric artery (arrow) of more than 180° and portal vein involvement (arrowhead)

**Fig. 2:**
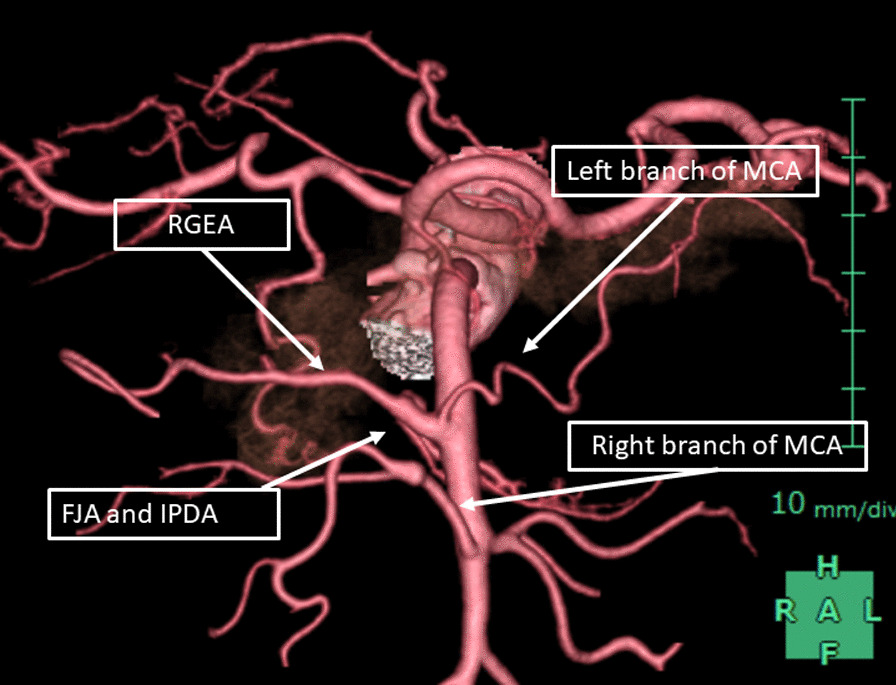
Three-dimensional constructed arterial imaging findings. The right gastroepiploic artery, left branch of the middle colic artery, first jejunal artery, and inferior pancreaticoduodenal artery formed a common trunk that branched from the right and slightly ventral side of the superior mesenteric artery

Contrast-enhanced computed tomography (CECT) revealed a hypovascular tumor in the uncinate process of the pancreas with tumor contact to the superior mesenteric artery (SMA) of more than 180° (approximately 190°) and with portal vein (PV) involvement, although there was no evidence of distant or lymph nodal metastasis (Fig. 1). Moreover, CECT revealed an arterial anomaly: the right gastroepiploic artery (RGEA), left branch of the middle colic artery (MCA), first jejunal artery (FJA), and inferior pancreaticoduodenal artery (IPDA) formed a common trunk that branched from the right and slightly ventral side of the SMA (Fig. 2). A diagnosis of adenocarcinoma was made by endoscopic ultrasound-guided fine needle aspiration. Thus, she was diagnosed with unresectable LA PDAC according to the Japan Pancreas Society classification [9] and immediately underwent four cycles of chemotherapy [gemcitabine plus nanoparticle albumin-bound paclitaxel (GnP)]. However, the lesion did not shrink and tumor contact with the SMA (approximately 190°) was not improved after chemotherapy. Because the probability of achieving curative resection was judged to be low, the patient received a radical dose [55.2 Gy (relative biological effectiveness (RBE) weighted absorbed dose) in 12 fractions] of CIRT with concurrent gemcitabine, and GnP therapy was continued.

**Fig. 3:**
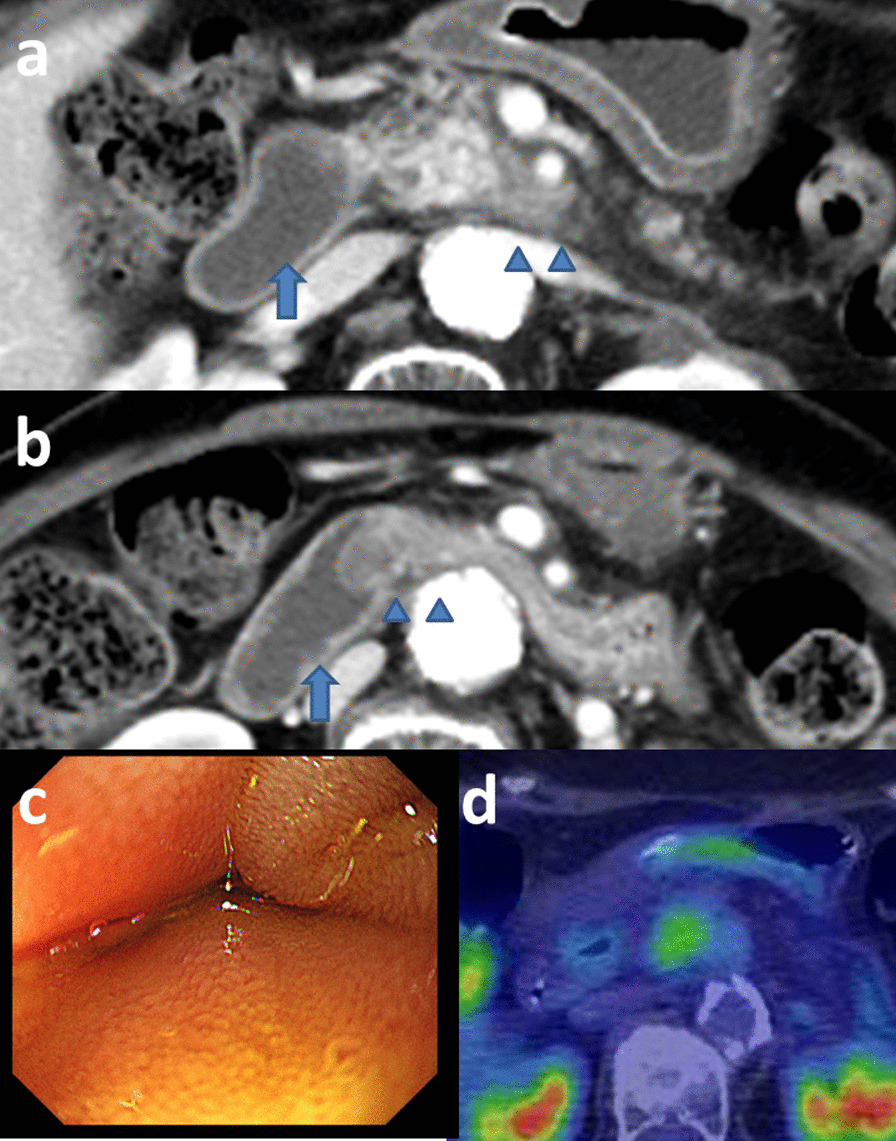
Findings of imaging studies after carbon-ion radiotherapy. **a** Contrast-enhanced computed tomography revealed no tumor growth (arrowheads). However, duodenal dilatation was observed (arrow). **b** CECT revealed a duodenal obstruction accompanied by wall thickening at the third portion of the duodenum (arrowheads). The oral duodenum was dilated (arrow). **c** Endoscopy revealed a duodenal compressive obstruction. The endoscope could not pass through this duodenal obstruction. **d** 18-fluoro-2-deoxy-glucose positron emission tomography/computed tomography after carbon-ion radiotherapy revealed slight FDG accumulation (standardized uptake value ranging from 3.14 to 3.20) in the pancreatic lesion without findings of distant or lymph nodal metastasis

**Fig. 4:**
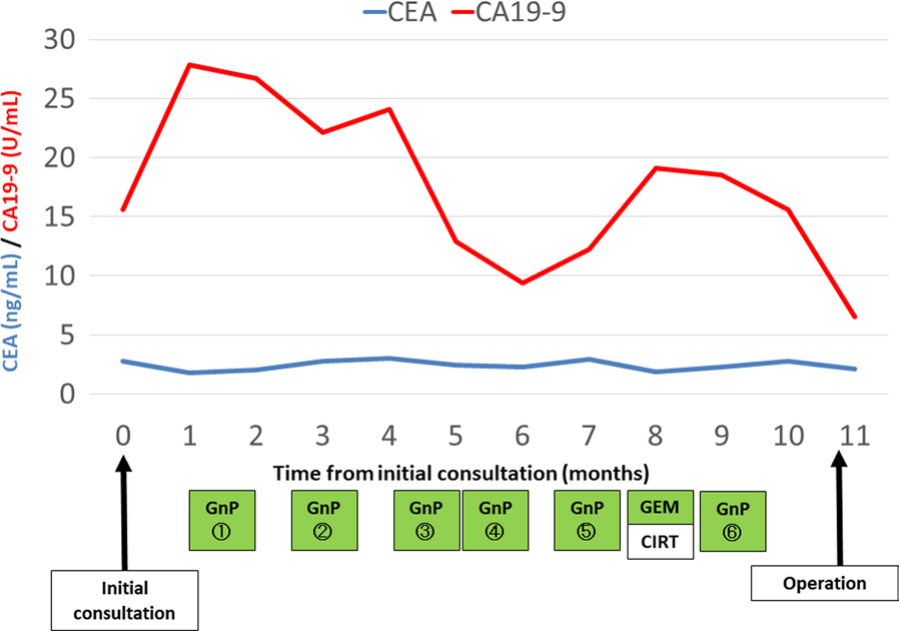
The progress of preoperative treatment and changes in tumor markers. Tumor marker concentrations were within the normal limits during the preoperative period. *CEA* carcinoembryonic antigen, *CA19-9* carbohydrate antigen 19-9, *GnP* gemcitabine plus nanoparticle albumin-bound paclitaxel, *GEM* gemcitabine, *CIRT* carbon-ion radiotherapy

She complained of vomiting about 2 months after CIRT. Although CECT showed no tumor growth and tumor contact with the SMA (approximately 190°) was not improved after CIRT, a duodenal obstruction accompanied by wall thickening at the third portion of the duodenum was observed (Fig. 3a and b). Endoscopy revealed a duodenal obstruction (Fig. 3c). She could not eat solid food and a trans-nasal feeding tube was inserted. The tip of feeding tube was placed at upper duodenum. 18-fluoro-2-deoxy-glucose positron emission tomography/computed tomography (FDG-PET/CT) revealed slight FDG accumulation in the pancreatic lesion without findings of distant or lymph nodal metastasis (Fig. 3d). The cause of the duodenal obstruction was thought to be an adverse effect of CIRT. Because a therapeutic intervention was needed to enable enteral nutrition, we proposed several treatment options, such as course observation with tube feeding, bypass surgery, or resection with the expectation of the anti-tumor effect of chemotherapy and CIRT, and she chose resection. Figure 4 shows the progress of the preoperative treatment and changes in tumor markers.

**Fig. 5:**
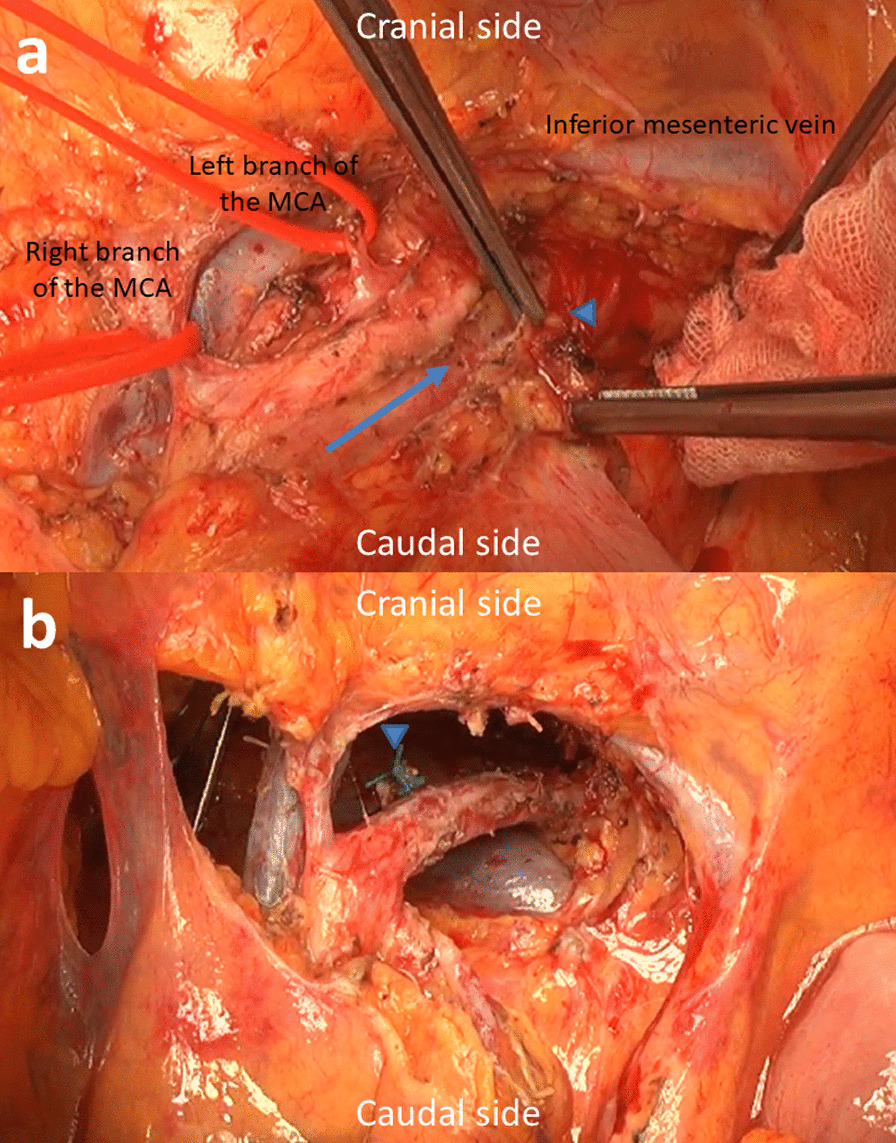
Intraoperative findings. **a** After incising the left side of the nerve plexus around the superior mesenteric artery (SMA) (arrow indicates the incision line), part of the nerve plexus was assessed as a frozen tissue section to confirm the absence of tumor cells (arrowhead). Right and left branches of the middle colic arteries (MCAs) are taped. **b** View after resection via incised transverse mesocolon. Arrowhead indicates the stump of the common trunk of the right gastroepiploic artery, left branch of the MCA, first jejunal artery, and inferior pancreaticoduodenal artery. The dorsal nerve plexus around the SMA was dissected by approximately 240°. The left renal vein was observed on the dorsal side of the SMA

Subtotal-stomach-preserving pancreatoduodenectomy with PV resection was performed (Fig. 5). Because nerve plexus dissection around the SMA was considered to be necessary to achieve R0 resection, a mesenteric approach was selected. Tissues around the pancreatic head region were slightly edematous and fibrotic because of CIRT; however, these were not too hard. After incising the transverse mesocolon, the SMA and superior mesenteric vein were identified, and the right branch of the MCA was taped (Additional file 1: Video S1). The ventral side of the SMA was exposed and the left side of the nerve plexus around the SMA was incised. Then, the left branch of the MCA was taped (Additional file 2: Video S2). Part of the nerve plexus around the SMA was assessed by means of a frozen tissue section to confirm the absence of tumor cells (Additional file 3: Video S3). Then, the SMA was taped (Additional file 4: Video S4). After ligating and cutting the root of the left branch of the MCA, the common trunk of the RGEA, left branch of the MCA, FJA, and IPDA, that branched from the right and slightly ventral side of the SMA, was taped (Additional file 5: Video S5). The cranial side of the SMA was taped and the left side of the SMA and the tumor were detached (Additional file 6: Video S6). After ligating and cutting the common trunk of the RGEA, the left branch of the MCA, FJA, and IPDA, dorsal side of the SMA and the tumor were detached (Additional file 7: Video S7). After exposing the first jejunal vein, the inferior pancreaticoduodenal vein was ligated and cut (Additional file 8: Video S8). Combined PV resection was performed, and the specimen was removed (Additional file 9: Video S9 and Additional file 10: Video S10). The surgical duration was 453 min and blood loss was 275 g.

**Fig. 6:**
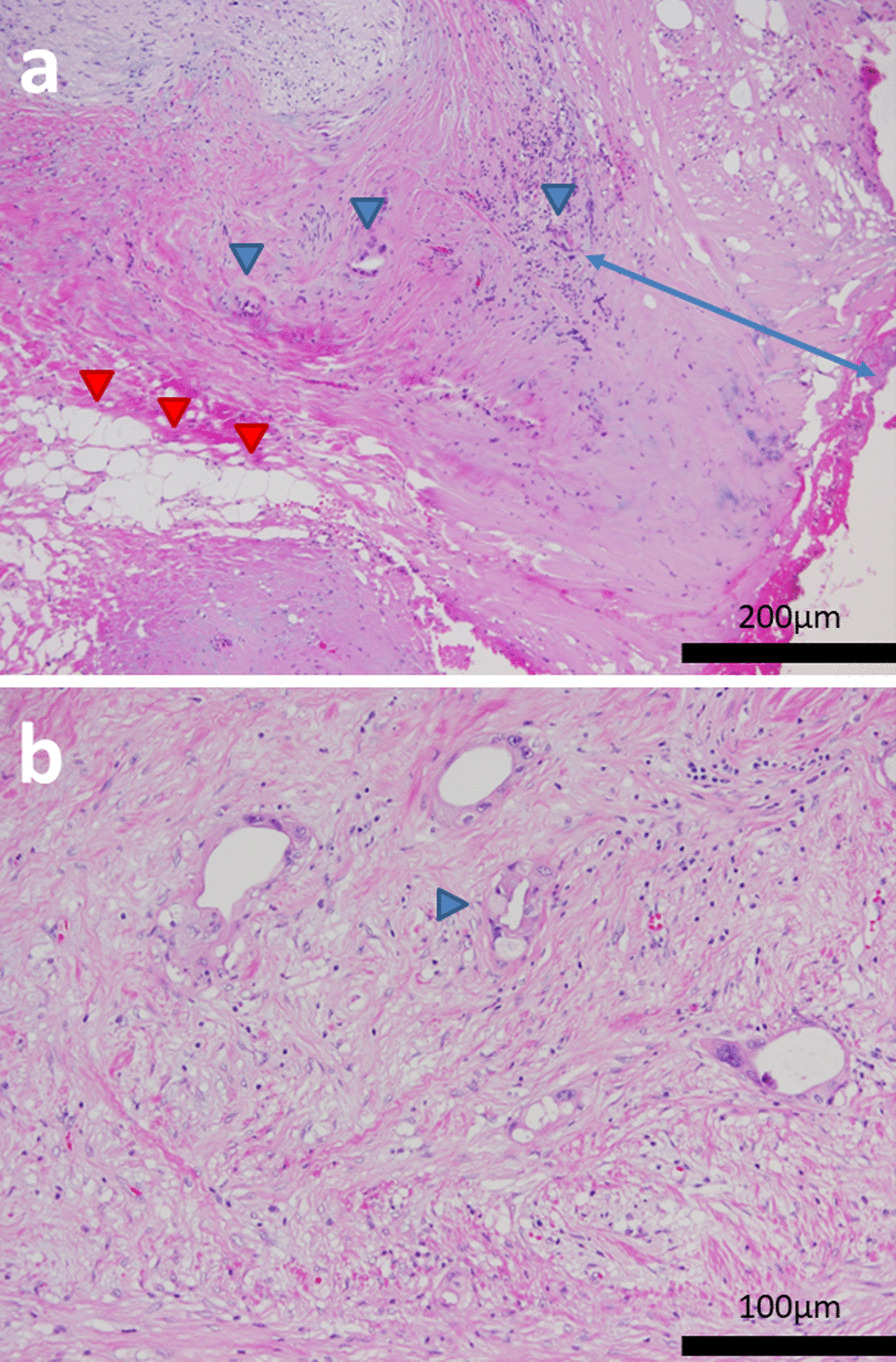
Microscopic findings of the resected specimen. **a** Viable tumor cells were observed in the replaced fibrotic tissues (blue arrowheads). Adipose degeneration was observed (red arrowheads). The distance of the dissected peripancreatic tissue margin was approximately 0.3 mm (blue arrow) and R0 resection was achieved. **b** Viable tumor cells with ductal formation were accompanied by vacuolar degeneration (arrowhead), and the effect grade was categorized as Evans grade IIA

Microscopically, viable tumor cells were observed in the replaced fibrotic tissues, and portal vein invasion and extrapancreatic nerve plexus invasion was observed; however, lymph nodal metastasis was not observed and R0 resection was achieved (Fig. 6a). Viable tumor cells with ductal formation were accompanied by vacuolar degeneration, and adipose degeneration was observed. The effect grade was categorized as Evans grade IIA (Fig. 6b). The duodenal wall showed acute inflammation and fibrosis without the presence of malignant cells.

Postoperatively, the patient recovered uneventfully except for controllable diarrhea and she was discharged on postoperative day 27. Postoperative adjuvant chemotherapy with tegafur/gimeracil/oteracil (S1) was administrated for 17 months. At the time of this report, 5 years have passed since the initial consultation and the patient has experienced no recurrence. Although examination for pancreatic function was not performed, her exocrine pancreatic disfunction was controllable with oral medication, and she did not develop diabetes during follow-up.

## Discussion

The resectability classification of PDAC proposes resectable, borderline resectable, and unresectable categories according to the probability of achieving an R0 resection with standard resection [9]. Unresectable PDAC because of local advancement has a high possibility of R2 resection with a macroscopic residual tumor because of vascular involvement [9]. The standard-of-care for unresectable LA PDAC is chemotherapy or chemoradiotherapy. Recently, intensive chemotherapeutic regimens such as the combination regimens of oxaliplatin, irinotecan, fluorouracil plus leucovorin (FOLFIRINOX), and GnP have been established as standards-of-care for unresectable PDAC. Although these regimens achieve excellent efficacy against PDAC, it remains difficult to achieve a cure by chemotherapy or chemoradiotherapy alone in patients with unresectable PDAC [3, 4]. However, recent advances in these therapies have enabled CS to be performed for selected patients with initially unresectable PDAC following favorable responses to preoperative treatment, and many studies have indicated that CS might extend the survival of patients with initially unresectable LA PDAC [5, 6]. However, there are many unresolved clinical questions concerning CS for initially unresectable PDAC. We experienced a long-term survival case with initially unresectable LA PDAC who underwent CS after chemotherapy followed by CIRT. The present case gives some insights into CS for unresectable LA PDAC.

Hammel et al. reported the results of an international, randomized clinical trial (LAP07), which evaluated whether chemoradiotherapy improved the overall survival of patients with unresectable LA PDAC controlled after 4 months of gemcitabine-based induction chemotherapy [10]. The median overall survival of patients randomized to receive chemotherapy alone and chemoradiotherapy was 16.5 months and 15.2 months, respectively. Therefore, the role of additional radiotherapy for unresectable LA PDAC is controversial. Chemoradiotherapy has also been administered as a preoperative treatment for PDAC. Chemoradiotherapy for unresectable LA PDAC is expected to achieve better local tumor control than chemotherapy alone, which may lead to a better R0 resection rate; however, the survival benefit of radiotherapy as a preoperative treatment remains unclear [11–13]. Currently, new radiation techniques, such as intensity-modulated radiotherapy, stereotactic body radiotherapy, and proton and carbon-ion radiotherapy are used as cancer treatments that deliver higher doses of radiation precisely to improve local control. Several studies have shown that these new radiotherapy techniques are effective at treating unresectable LA PDAC, with a positive relationship between increased dose and clinical outcomes [14–16]. However, the radiation dose that can be delivered to PDAC is limited because there are many radiosensitive organs around the pancreas. CIRT is a unique external-beam radiotherapy that offers better dose conformity and higher biological effectiveness than conventional photon therapy or proton therapy [17]. Moreover, the effectiveness of CIRT is known to be relatively independent of the oxygenation of the irradiated tissue [18] and the underlying cell cycle phase [19]. Thus, CIRT may have the potential to offer a higher probability of tumor control and survival benefit for PDAC than conventional radiotherapy. Several Japanese studies reported the favorable survival outcomes of combined CIRT and mainly gemcitabine-based chemotherapy for unresectable LA PDAC with median overall survival ranging from 21.5 to 25.1 months [7, 8]. These results suggest an advantage of CIRT over conventional radiotherapy. However, it is still far from being curative. Therefore, a combination of CIRT and recent chemotherapy regimens, such as FOLFIRINOX or GnP, or CIRT as a preoperative treatment might be a candidate to improve the clinical outcomes of unresectable LA PDAC. The present case was judged as unresectable after GnP therapy and underwent radical CIRT. However, because a therapeutic intervention was needed to enable enteral nutrition after CIRT, the patient underwent surgery, and consequently, long-term survival was achieved. This suggests that multidisciplinary treatment consisting of a combination of recent chemotherapy and CIRT may be beneficial for unresectable LA PDAC.

The morbidity and mortality rate of CS after chemotherapy or chemoradiotherapy for initially unresectable PDAC was reported to be comparable with that of conventional pancreatectomy [20, 21]. However, the surgical difficulty and safety of preoperative CIRT are of concern. Shinoto et al. reported a Phase I trial of CIRT as preoperative treatment for resectable PDAC [22]. Twenty-six patients with resectable PDAC were irradiated per dose-escalating protocol from 30 Gy (RBE) to 36.8 Gy (RBE) in eight fractions. Resection was finally performed in 21 patients and the median time from the last day of CIRT to resection was 20 days (range, 14–30 days). Although the surgical difficulty and safety of preoperative CIRT were not mentioned in detail, they reported some small changes including fibrosis after CIRT; however, the influence of the surgical maneuver was minimal. The present case underwent a radical dose of CIRT [55.2 Gy (RBE)] followed by surgery about 2.5 months after the last day of CIRT. However, the surgical difficulty of CIRT was not increased in the present case, although tissues around the pancreatic head region were slightly edematous and fibrotic. Because carbon-ions can be concentrated to a localized lesion, damage to normal tissue is reduced and the rate of gastrointestinal grade 3 toxicity after CIRT for unresectable LA PDAC was reported to range from 1 to 3% [7, 8], although a duodenal obstruction was observed in the present case. These facts may indicate the feasibility of CIRT as an option for the preoperative therapy of unresectable LA PDAC.

It was reported that 55.6%–83.3% of IPDA forms a common trunk with the jejunal artery and 65.6% of the IPDA exists on the dorsal side of the SMA [23]. When performing resection for LA PDAC invading the dorsal side of the SMA, how to transect IPDA is important to perform safe procedure and achieve an R0 resection. In the present case, there was an arterial anomaly, which allowed the common trunk of the RGEA, left branch of the MCA, FJA, and IPDA, to be safely transected away from the tumor and the R0 resection could be performed. Her arterial anomaly might explain her long-term survival.

Indicators of patient selection for CS for initially unresectable LA PDAC are unknown. According to recent studies, most patients who underwent CS had no evidence of a response on imaging and there was no survival difference between responders and non-responders [13, 24–27]. Moreover, the accuracy of CECT to determine resectability was significantly decreased after preoperative treatment [24, 25]. These studies and the present case suggest that an indication of CS for initially unresectable LA PDAC cannot be determined by imaging responses only. Measuring CA19-9 concentrations may be a useful predictor of survival, and the normalization or marked decrease in CA19-9 concentration following preoperative treatment might correlate with better survival [27, 28]. Although there are no consensus criteria for CA19-9 concentrations on which to base the selection of patients who could benefit from CS, the fact that the present patient’s tumor marker concentrations were within normal limits during the preoperative period might explain the patient’s long-term survival.

## Conclusions

We experienced the long-term survival of a case with initially unresectable LA PDAC who underwent CS after chemotherapy followed by CIRT. The present case suggests that multidisciplinary treatment consisting of a combination of recent chemotherapy and CIRT may be beneficial for unresectable LA PDAC. However, further studies are required to assess the true efficacy of this treatment strategy.

### Supplementary Information

**Additional file 1:** Operative procedure 1.

**Additional file 2:** Operative procedure 2.

**Additional file 3:** Operative procedure 3.

**Additional file 4:** Operative procedure 4.

**Additional file 5:** Operative procedure 5.

**Additional file 6:** Operative procedure 6.

**Additional file 7:** Operative procedure 7.

**Additional file 8:** Operative procedure 8.

**Additional file 9:** Operative procedure 9.

**Additional file 10:** Operative procedure 10.

## Data Availability

Not applicable.

## Abbreviations

PDAC: Pancreatic ductal adenocarcinoma
LA: Locally advanced
CS: Conversion surgery
CIRT: Carbon-ion radiotherapy
CEA: Carcinoembryonic antigen
CA19-9: Carbohydrate antigen 19-9
CECT: Contrast-enhanced computed tomography
SMA: Superior mesenteric artery
PV: Portal vein
RGEA: Right gastroepiploic artery
MCA: Middle colic artery
FJA: First jejunal artery
IPDA: Inferior pancreaticoduodenal artery
GnP: Gemcitabine plus nanoparticle albumin-bound paclitaxel
RBE: Relative biological effectiveness
FEG-PET/CT: 18-Fluoro-2-deoxy-glucose positron emission tomography/computed tomography
FOLFIRINOX: Oxaliplatin, irinotecan, fluorouracil plus leucovorin
